# A Replication-Defective Myxoma Virus Inducing Pro-Inflammatory Responses as Monotherapy and an Adjuvant to Chemo- and DC Immuno-Therapy for Ovarian Cancer

**DOI:** 10.3390/v17081058

**Published:** 2025-07-29

**Authors:** Martin J. Cannon, Jia Liu

**Affiliations:** Department of Microbiology and Immunology, University of Arkansas for Medical Sciences (UAMS), Little Rock, AR 72205, USA; cannonmartin@uams.edu

**Keywords:** oncolytic virus, myxoma virus, immunoregulatory, immunotherapy platform, host range factor, type 1 interferon, type 1 interferon stimulated genes, proinflammatory

## Abstract

Myxoma virus (MYXV), a rabbit-specific poxvirus and non-pathogenic in humans and mice, is an excellent candidate oncolytic virus for cancer therapy. MYXV also has immunotherapeutic benefits. In ovarian cancer (OC), immunosuppressive tumor-associated macrophages (TAMs) are key to inhibiting antitumor immunity while hindering therapeutic benefit by chemotherapy and dendritic cell (DC) vaccine. Because MYXV favors binding/entry of macrophages/monocytes, we examined the therapeutic potential of MYXV against TAMs. We found previously that a replication-defective MYXV with targeted deletion of an essential gene, *M062R*, designated Δ*M062R* MYXV, activated both the host DNA sensing pathway and the SAMD9 pathway. Treatment with Δ*M062R* confers therapeutic benefit comparable to that of wild-type replicating MYXV in preclinical models. Here we found that Δ*M062R* MYXV, when integrated with cisplatin and DC immunotherapy, further improved treatment benefit, likely through promoting tumor antigen-specific T cell function. Moreover, we also tested Δ*M062R* MYXV in targeting human immunosuppressive TAMs from OC patient ascites in a co-culture system. We found that Δ*M062R* treatment subverted the immunosuppressive properties of TAMs and elevated the avidity of cytokine production in tumor antigen-specific CD4^+^ T cells. Overall, Δ*M062R* presents a promising immunotherapeutic platform as a beneficial adjuvant to chemotherapy and DC vaccine.

## 1. Introduction

Cancer cells can cultivate and maintain the tumor environment to escape from host immune detection and elimination [1]. Once the tumor environment is established, cancer cells often develop resistance and tolerance to repeated chemotherapy treatments, which eventually leads to irreversible progression in cancer cell growth and lethality in patients [2]. This is often the case for ovarian cancer (OC) patients [3]. Ovarian cancer is a leading cause of cancer death in women, with over 200,000 deaths annually worldwide [4]. While platinum-based chemotherapy remains the standard care for OC patients and most patients initially respond, chemo-resistance arises frequently [5,6]. High-grade serous OC (HGSOC) is the most common form of OC in women (over 70%) [3], and parameters indicating an immunosuppressive tumor environment suggest a poor prognosis [7,8]. Targeting key elements of the OC immunosuppressive tumor environment, such as tumor-associated macrophages (TAMs) [9] and Tregs [10], leads to the development of novel therapeutics, for instance, through the use of dendritic cell (DC) vaccines [11,12]. In this study, we considered the promise of DC vaccines in treating immunosuppressive HGSOC and aimed to develop a combinatorial treatment strategy to further advance the therapeutic benefit.

Myxoma virus (MYXV) belongs to the poxvirus family and has a unique safety profile for therapeutic applications in humans and in preclinical mouse models [13]. These viruses also have a large genome capacity to allow extensive genetic engineering for therapeutic molecules, such as IL-12 [14], IL-15 [15], and TNF [16], to edit regional and systemic immune networks. Because WT MYXV has oncolytic properties by specifically infecting and killing cancer cells [17] and engineered mutant MYXVs can also activate host innate immune signaling [16,18,19], this virotherapy vector represents a novel approach to eliminate tumor cells while reprogramming the tumor environment for customized therapy. Besides utility as monotherapy, MYXV has versatility as a complement for other therapeutics, e.g., chemotherapy (cisplatin [20] and gemcitabine [21]). In this study, we further explore the potential application of mutant MYXV for immunotherapy and as an adjuvant to chemotherapy and DC vaccination.

Although replicating oncolytic viruses can amplify killing of tumor cells, antiviral innate immunity often rapidly eliminates their presence in the tumor environment [22]. However, despite their removal, their immunological impact of oncolytic viruses may continue to provide therapeutic benefit [23]. The debate on whether replication competency determines the longevity and effectiveness of therapeutic immune responses continues. Our findings further support the therapeutic potential of replication-defective viruses.

In this study, we tested MYXV virotherapy combined with cisplatin chemotherapy and a Th17-inducing DC vaccine that has recently shown promise for treatment of advanced-stage ovarian cancer patients [24]. We show that combinatorial treatments with MYXV improve survival in a mouse model of OC when compared with cisplatin or DC monotherapy. We further show that MYXV can alleviate immune suppression by patient-derived ovarian tumor-associated CD14^+^ macrophages (TAMs) in coculture with tumor antigen-specific CD4^+^ T cells.

## 2. Materials and Methods

### 2.1. Human Subjects

Deidentified ovarian cancer patient samples were provided by the Women’s Oncology Clinic, Winthrop P. Rockefeller Cancer Institute, University of Arkansas for Medical Sciences (UAMS) and the Mayo Clinic Ovarian Cancer Research Program Biospecimens Bank under IRB-approved protocols. Ovarian tumor ascites samples were recovered at the time of surgery.

### 2.2. Cell Culture and Virus Stock

Patient ovarian cancer cells (KAJ343, OvCa-37, and OvCa-43) [20] were cultured in RPMI complete growth medium supplemented with glutamine, penicillin/streptomycin and 10% fetal bovine serum. KAJ343 and OvCa-43 were high-grade serous ovarian carcinomas. OvCa-37 underwent neoadjuvant chemotherapy, and diagnosis was based on paracentesis ascites rather than surgical pathology, which was not defined. BSC40 (ATCC CRL-2761), ID8 TP53^−/−^ (provided by Iain McNeish, Imperial College, London, UK) [25], and ID8-SP17 cells (provided by Keith Knutson, Mayo Clinic, Jacksonville, FL, USA) [26] are cultured in DMEM complete medium. The complete growth medium (e.g., DMEM Lonza/BioWhittaker Catalog no 12-604Q) was supplemented with 10% FBS (Atlanta Biologicals, Minneapolis, MN, USA), 2 mM glutamine (Corning Cellgro, Millipore Sigma, St. Louis, MO, USA), and 100 μg per mL of Pen/Strep (Corning Cellgro, Millipore Sigma, St. Louis, MO, USA); for RPMI1640 complete culture medium, in addition to FBS, glutamine, and Pen/Strep, 2-mercaptoethanol (MP Biomedicals, Solon, OH, USA) was supplemented to a final concentration of 0.05 mM.

The viruses used were originally derived from the Lausanne strain of myxoma virus (MYXV) (GenBank Accession NC_001132.2/AF170726.2). The MYXV *M062R* deletion mutant (Δ*M062R*) and WT MYXV have been described previously [27]. Myxoma virus stocks were prepared on BSC-40 cells and purified with a sucrose step gradient through ultracentrifugation as previously described [28].

### 2.3. RT Realtime (RT^2^) PCR

Human primary patient cancer cells KAJ434, OvCa-37 and OvCa-43 or murine ID8 derivatives ID8-TP53^−/−^ were seeded the day before. The next day cells were mock treated or infected at an moi of 5 for either WT or Δ*M062R* MYXV [27]. At 24 h post-infection, cells were harvested with the Direct-zol RNA Mini Prep kit (catalog # R2052, Zymo, Irvine, CA, USA) according to the manufacturer’s standard protocol. RNA quality was examined by running on the RNA gel to check 28S and 18S integrity and photospectrometer to estimate concentration. Equal amounts of total RNA in a maximal volume of 6 μL were used for cDNA synthesis using the NEB PhotoScript^®^ First Strand cDNA Synthesis Kit (Catalog # E6300L, NEB Inc., Ipswich, MA, USA) as instructed in the manufacturer’s standard protocol. Realtime PCR was conducted following manufacturer standard protocol (Luna Universal qPCR Master Mix, NEB Inc., Ipswich, MA, USA). The SYBR green RT-PCR primers used in this study are listed in Table 1.

### 2.4. Tumor Model Establishment and Treatment

The animal study was approved by the UAMS Institutional Animal Care and Usage Committee (IACUC) at the University of Arkansas for Medical Sciences (UAMS). ID8-TP53^−/−^ and ID8-SP17 ovarian tumor cell lines were used for mouse model studies. The ID8-SP17 cell line was kindly provided by Dr. Keith Knutson, Mayo Clinic, Jacksonville, FL, USA. The tumor models have been previously described [25,26]. Briefly, 4 studies were performed. (a) The “WT MYXV+ cisplatin+ DC vaccine” study. 3 × 10^6^ ID8-TP53^−/−^ tumor cells were injected intraperitoneally (i.p.), followed by WT MYXV treatment (10^8^ pfu, i.p.) given every 2 days for 4 doses, starting on day 7 after tumor cell engraftment. Cisplatin (3 mg/Kg, i.p.) was given every 3 days for 3 doses starting on day 16. DC vaccine (10^6^ cells, i.p.) was administered every 7 days for 4 doses, starting on day 30. (b) The “late Cisplatin + Δ*M062R* + DC vaccine” study with the ID8-TP53^−/−^ model. Cisplatin (3 mg/Kg, i.p.) was given every 3 days for 3 doses starting on day 16 after tumor cell engraftment. Δ*M062R* treatment (10^8^ pfu, i.p.) was given every 2 days for 4 doses, starting on day 28. DC vaccine (10^6^ cells, i.p.) was administered every 7 days for 4 doses, starting on day 44. (c) The early cisplatin + Δ*M062R* + DC vaccine study with the ID8-TP53^−/−^ model. Cisplatin (3 mg/Kg, i.p.) was given every 3 days for 3 doses starting on day 7 after tumor cell engraftment. Δ*M062R* treatment (10^8^ pfu, i.p.) was given every 2 days for 4 doses, starting on day 20. DC vaccine (10^6^ cells, i.p.) was administered every 7 days for 4 doses, starting on day 35. (d) The ID8-SP17 HGSOC model. Tumor cells were injected i.p. at 10^6^ cells/mouse, and cisplatin (1 mg/Kg, i.p.) was given every 3 days for 3 doses starting on day 7. Δ*M062R* treatment (10^8^ pfu, i.p.) was first given on day 19 and followed by DC vaccine on day 21. This Δ*M062R*-DC vaccine mini-regimen was repeated every 7 days for 4 rounds in total.

### 2.5. DC Vaccine Preparation

Mouse bone marrow cells (2 × 10^6^/mL) were cultured in RPMI 1640 medium plus 10% fetal bovine serum (RPMI/10) with GM-CSF, IL-4, IL-15 and a p38 MAPK inhibitor (Calbiochem/EMD Chemicals). On days 3 and 5, half the medium was removed and replaced with RPMI/10 plus the same concentration of cytokines and p38 MAPK inhibitor. Maturation cytokines (PGE2, TNFα and IL-1β) and murine Sp17 peptides (32 mer, overlapping by 10 amino acids, covering residues 1-144, 50 μg/mL) were added on day 5, and the DC was harvested on day 7. Cytokine and reagent concentrations are as described for human Th17-DC [33].

### 2.6. Human Ovarian Cancer Ascites CD14^+^ Macrophages and CD4^+^ T Cell Co-Culture System

CD14^+^ TAMs were recovered from primary ovarian tumor ascites by CD14^+^ microbead and magnetic column purification (Miltenyi Biotec, Germany). Human CD4^+^ T cells specific for ovarian tumor peptide antigens were derived through stimulation with TADG14v peptide-loaded Th17-inducing dendritic cells, as previously described [33,34,35]. CD14^+^ ascites TAMs were either uninfected or infected with WT or Δ*M062R* MYXV (at a moi of 10, pre-incubated on ice for 1 h, then washed one time with PBS). Ascites CD14^+^ TAMs (5 × 10^5^/well) were then co-cultured with ovarian tumor antigen-specific CD4^+^ T cells (5 × 10^5^/well) in 0.7 mL RPMI plus antibiotics, 5 × 10^−5^ M 2-mercatopethanol and 10% human AB serum (RPMI 10Hu). After 48 h, tumor antigen peptide-loaded and irradiated (75 gray) autologous lymphoblastoid cells (2.5 × 10^5^ cells/well) were added in 0.2 mL RPMI/10Hu, and the cocultures were incubated o/n in the presence of Brefeldin A. Where indicated, cocultures were treated with a STING inhibitor (1 μM H-151) or a TBK-1 inhibitor (2 μM BX-795). Following coculture, CD4^+^ T cells were stained with PE-anti-CD4 antibody, fixed and permeabilized with fixation/permeabilization buffer (Affymetrix eBioscience, San Diego, CA, USA) and stained with APC anti-TNF antibody. Samples were analyzed with a FACSCalibur flow cytometer (BD, Franklin Lakes, NJ, USA) and CellQuestPro software (Version 5.1.1.1, BD, Franklin Lakes, NJ, USA).

### 2.7. Statistical Analyses

GraphaPrism 10.1 was used for statistical analyses. Survival study was performed using Kaplan–Meier analyses followed by Long-rank comparison. Statistical significance is defined by * *p* < 0.05.

## 3. Results

### 3.1. Myxoma Virus Infection in Primary Cells of Human Ovarian Cancer Environment Stimulated Proinflammatory Gene Expression and Up-Regulation of Sp17

**Figure 1:**
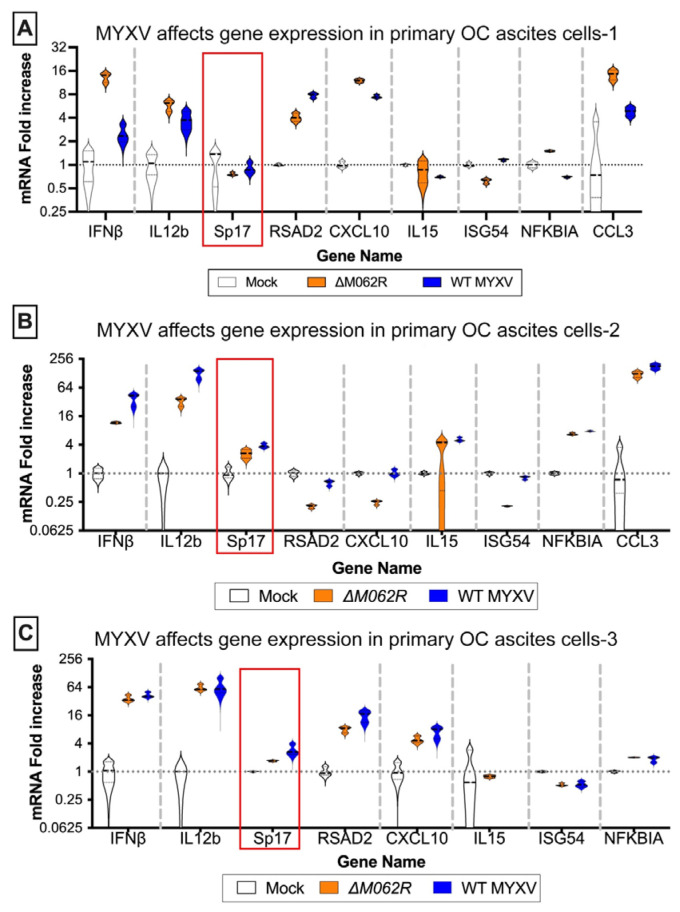
**Myxoma virus treatments of primary human ovarian cancer ascites cells induce proinflammatory molecule expression and upregulation of Sp17.** Ascites cells from OC patients, (**A**) OvCa37, (**B**) KAJ343, and (**C**) OvCa43, were mock treated or infected with MYXV, WT or ΔM062R, at an MOI of 5 for 18 h before cells were harvested for RNA extraction and RT^2^-PCR to assay target gene expression levels for semi-quantitative comparison. The red frame marks the ovarian cancer cell-specific antigen, Sp17, expression levels.

**Figure 2:**
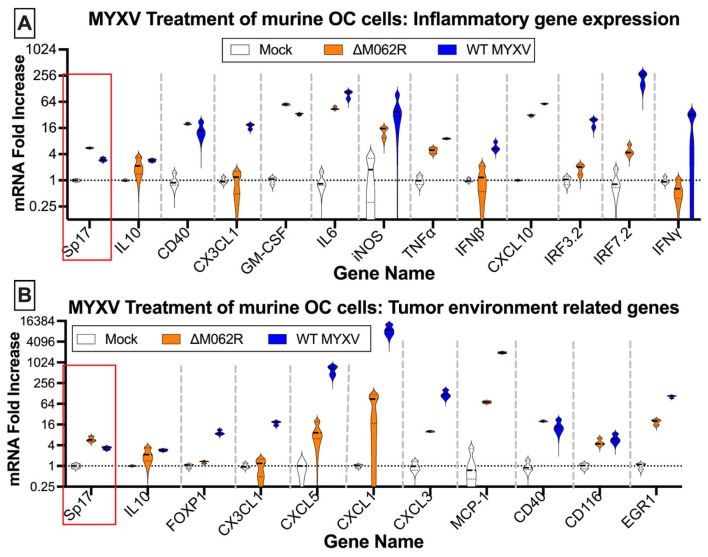
**Myxoma virus treatments of murine ID8 TP53^−/−^ induce inflammatory gene expression and alter gene expression affecting the tumor environment.** Murine OC cells were mock treated or infected with MYXV at an MOI of 5 for 18 h before RNA extraction and RT^2^-PCR. Genes related to inflammatory responses (**A**) and contributing to tumor environment maintenance (**B**) were examined for their expression affected by MYXV treatments. In either array, OC tumor marker, murine Sp17, was also examined.

Antigen Sp17 is a signature protein found in OC cells [36,37]. Expression of Sp17, however, is not found in normal female cells; thus, Sp17 antigen may be an excellent target for OC immunotherapy [37,38,39]. We included Sp17 in our RT-PCR array as an indicator for cancer cells. Two MYXV viruses, a replicating WT and replication defective mutant virus (Δ*M062R*), were used for the study. Primary OC patient ascites cells from three patients were mock treated or treated with MYXV at an moi of 5-10 before examining the gene expression profile. MYXV treatment increased Sp17 expression (Figure 1A–C, red frame) in addition to up-regulation of IFNβ and other proinflammatory molecules (e.g., IL12b and CXCL10) (Figure 1). We performed similar tests using the murine OC cell line ID8-TP53^−/−^ that was used for establishing a syngeneic OC model system as previously described [25]. We found that MYXV treatment increased expression of proinflammatory molecules (Figure 2A), similar to what we saw in human cells (Figure 1). Moreover, Sp17 expression is also up-regulated (Figure 2A,B, red frame), similarly as found in human primary ovarian tumors (Figure 1). These observations suggest that MYXV treatment may enhance the immunogenicity of ovarian tumor cells and render them more sensitive to SP17-targeted DC vaccination. Using an array panel to examine gene expression that shapes a pro-cancer tumor environment, we found that Δ*M062R* treatment moderately altered gene expression to a lesser degree than wildtype (WT) MYXV (Figure 2B). In our previous study, in human macrophages Δ*M062R* treatment stimulates proinflammatory gene expression at both RNA and protein levels, while WT MYXV infection seems to promote immunosuppressive gene expression [18]. Considering cells examined in this study are tumor cells or from the tumor environment, our finding provides new insights into cellular responses from cancer cells to infections by these two viruses.

### 3.2. Wildtype MYXV Treatment Preceding Cisplatin Treatment Led to Moderate Therapeutic Benefit in a High Grade Serious Ovarian Cancer (HGSOC) Murine Syngeneic Model

**Figure 3:**
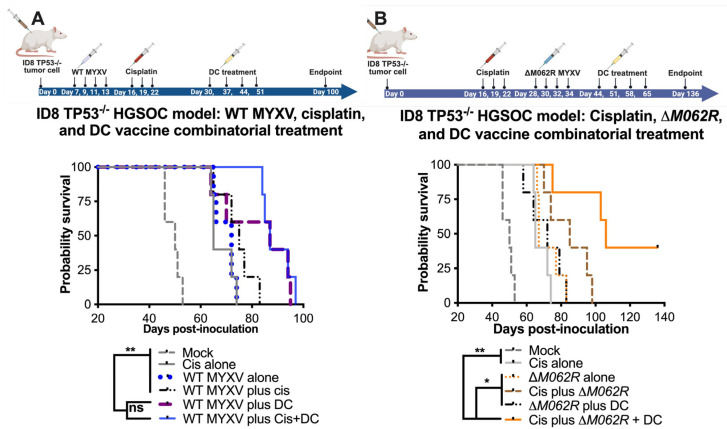
**Myxoma virus treatments facilitate survival in the combinatorial treatment with cisplatin and DC vaccine.** C57/BL6 mice were injected through the intraperitoneal route (i.p.) with 3 × 10^6^ ID8-TP53^−/−^ cells on day 0 and randomly grouped into 5 mice per treatment. Cisplatin was injected at 3 mg/kg i.p. on days 16, 19 and 22. WT-MYXV (10^8^ pfu, i.p.) (**A**) was injected on days 7, 9, 11 and 13 (before cisplatin treatment), while Δ*M062R* MYXV (10^8^ pfu i.p.) (**B**) was injected on days 28, 30, 32 and 34 (following cisplatin treatment). Sp17 peptide-loaded DC (10^6^) were injected subcutaneously (s.c.) on days 30, 37, 44 and 51 for WT-MYXV recipients and days 44, 51, 58 and 65 for Δ*M062R* MYXV recipients. The log-rank (Mantel–Cox) test was conducted for Kaplan–Meier survival comparison. * *p* < 0.05, ** *p* < 0.001, ns: not significant.

The recent success of a DC vaccine trial in OC patients provided exciting and novel options targeting the unique ovarian tumor environment [24]. With both human and murine Sp17 expressed in OC cells, we proposed a proof-of-concept DC vaccination strategy of pulsing Sp17 peptide on DCs for the in vivo study as previously described [26]. In our previous study, we found that WT MYXV treatment before cisplatin treatment provided better therapeutic benefit than either WT MYXV monotherapy or WT MYXV provided after cisplatin treatment [20]. The ability of replicating oncolytic WT MYXV to eliminate OC cells facilitated cisplatin control of tumor progression, hence the rationale of our combination treatment strategy. Thus, with a replicating oncolytic agent promoting the expression of tumor antigen, we tested whether a DC vaccine may be a good option to prolong survival further. We indeed found that WT MYXV and DC provided a significant improvement of survival with and without cisplatin treatment (Figure 3A) with a median survival of 87 days for either regime. Mock treatment, cisplatin alone, WT MYXV alone, and WT MYXV plus cisplatin show a median survival of 50, 65, 72, and 75 days, respectively.

### 3.3. M062R-Null (ΔM062R) MYXV Monotherapy or Combined with DC Vaccine Improved Survival in Murine Syngeneic HGSOC Models

Due to the association of the Sp17 antigen in OC with tumorigenicity, tumor progression, chemoresistance, and immunosuppressiveness, Sp17 becomes a promising therapeutic target for vaccine development in treating OC [37,38,40,41]. In our study, the DC vaccine is indeed designed to target the antigenic epitopes of Sp17 [42].

In our previous study [20], we examined the therapeutic potential of a replication-defective MYXV called *M062R*-null MYXV (Δ*M062R*) as either monotherapy or in combination with chemotherapy. We found previously that Δ*M062R* treatment after cisplatin provided better survival [20]. Recently, we further examined the immunostimulatory effect of Δ*M062R* and found this virus superior in inducing proinflammatory responses in monocytes/macrophages by triggering DNA-sensing stimulated type 1 interferon responses (IFN-I) [18]. The effect induced by Δ*M062R* in myeloid cells is different from the conventional DNA-sensing stimulated IFN-I responses and promotes a potent proinflammatory outcome [18,43], indicating an additional mechanism governing the unique immunological state [43]. Thus, besides its being a potential adjuvant to cisplatin, we examined if Δ*M062R* has therapeutic potential for immunotherapy and may also be an adjuvant for DC vaccine. In the clinical trial of the DC vaccine, all the patients were treated after platinum-based therapy and tumor debunking, thus we did not specifically examine the DC vaccine as a monotherapy in our study. The ID8-TP53^−/−^ syngeneic model system portrays the conditions of HGSOC in humans [44], and it presents an aggressive form of OC (Figure 3). We found that combined treatment with cisplatin, Δ*M062R*, and DC vaccine led to significant improvement in survival (*p* < 0.0001, Long-rank Mantel–Cox test, with a median survival of 106 days, Figure 3B) compared to mock treatment, cisplatin alone, and Δ*M062R* alone, with median survival of 50, 65, and 67 days, respectively. Moreover, while combination treatment of the cisplatin-Δ*M062R* or Δ*M062R*-DC vaccine noticeably improved median survival compared to monotherapy with median survival of 85 and 72 days (not reaching statistical significance), respectively, the cisplatin-Δ*M062R*-DC vaccine combinatorial regime is significantly better than the other two-regime treatments (*p* < 0.0197 and *p* < 0.0127, respectively, by the Long-rank Mantel–Cox test). In this study, however, we did not evaluate the therapeutic effect of the DC vaccine in facilitating cisplatin chemotherapy or Δ*M062R* virotherapy. We also did not examine whether the Δ*M062R* therapeutic benefit is synergistic/additive based on the same mechanism as the DC vaccine. We recognized that in this study the treatment of cisplatin or virotherapy was provided at a much later time, which was from days 16 or 28, respectively (see Figure 3B diagram on treatment schedule), than earlier studies of starting treatment at 7 days post-tumor establishment [20]. Although there is little evidence suggesting that a moderate delay in treatment would affect overall patient survival [45,46], we decided to investigate whether starting Δ*M062R* virotherapy or DC vaccine early in the combinatorial treatment offers any therapeutic benefit. We thus designed a follow-up study using the same ID8-TP53^−/−^ syngeneic model system that will be discussed next.

### 3.4. M062R-Null (ΔM062R) MYXV Treatment in Combination with DC Vaccine Targeting Sp17 Antigen Significantly Improved the Treatment Outcome of Cisplatin

**Figure 4:**
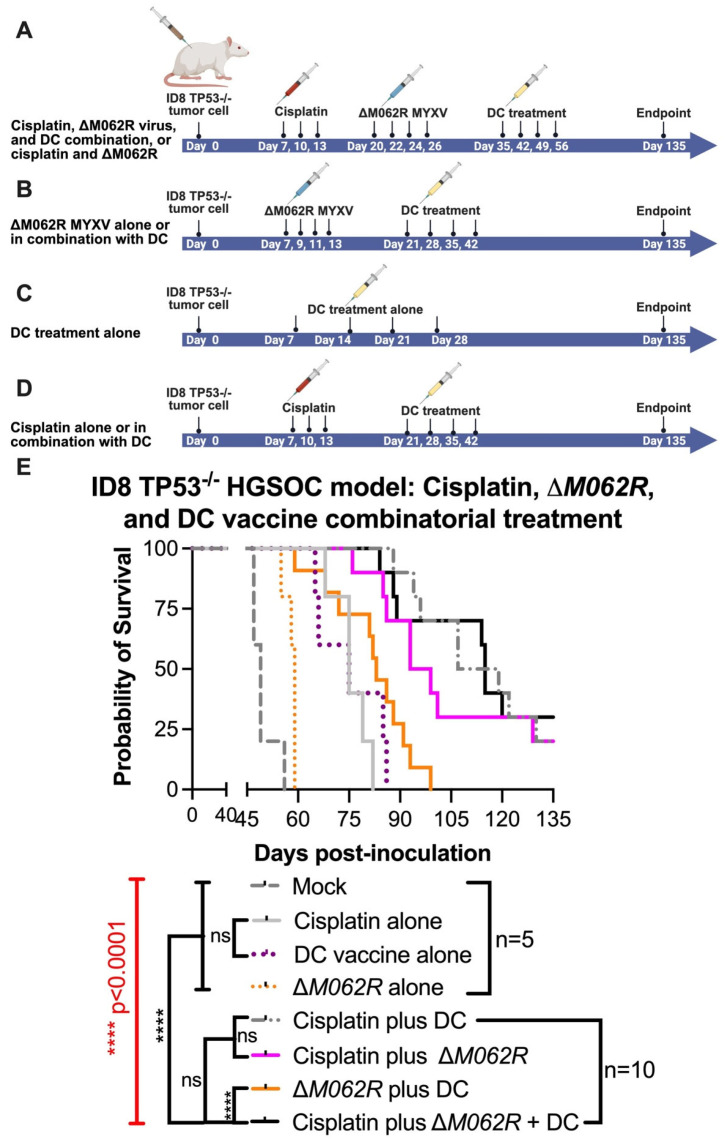
**Treatment of Δ*M062R* further enhances the therapeutic effect of the DC vaccine and cisplatin in a combinatorial treatment.** C57/BL6 mice were injected i.p. with 3 × 10^6^ ID8-TP53^−/−^ cells on day 0. Five mice were randomly assigned per group for mock and the individual treatment controls, and 10 mice were assigned per group for double or triple combinatorial treatments. Regime design is shown in (**A**) for cisplatin plus Δ*M062R* and the triple combinatorial treatment. (**B**). Treatment design for Δ*M062R* alone or the combination of Δ*M062R* and DC vaccine. Treatment schedule of DC vaccine alone is shown in (**C**). Treatment schedule of cisplatin alone or cisplatin plus DC vaccine schedule is shown in (**D**). In general, treatment starts 7 days after tumor establishment. Treatment with Δ*M062R* is at 10^8^ pfu, i.p., every 2 days for a total of 4 injections. Cisplatin was injected i.p. at 3 mg/Kg every 3 days for a total of 3 treatments. DC vaccine is injected s.c. 8–9 days after the previous treatment (if it is a combination treatment) every 7 days for a total of 4 vaccinations. (**E**). The Kaplan–Meier survival curve is shown, and Δ*M062R* plus DC provided a superior therapeutic outcome with or without cisplatin. The log-rank (Mantel–Cox) test was conducted with statistical significance defined as the following, **** *p* < 0.0001, and ns: not significant.

In this second stage of study, we modified the treatment schedules of the corresponding controls as shown in the diagram of Figure 4. We focused on the logistics of therapy design similar to a clinical setting and followed the following general principles: (1) to start the first treatment at 7 days post-tumor establishment, and (2) to start the DC vaccine at 8–9 days after the prior treatment for combination treatments. Although introducing Δ*M062R* treatment early as the first course of therapy (median survival of 59 days, *p* = 0.0071, Kaplan–Meier analysis by Long-rank Mandel–Cox test) significantly improved the survival compared to the mock-treated group (median survival of 49 days), it did not provide a dramatic edge to prolong survival over either cisplatin alone (median 75, *p* = 0.0019) or DC vaccine alone (median 75, *p* = 0.0019). This is consistent with our previous finding on the utility and timing of Δ*M062R* [20]. We also found that as a monotherapy, early initiation of DC vaccine (median survival of 75 days) is as effective as cisplatin (median survival of 75 days) at the same time frame in this model (no statistically significant difference). Although two-regime and three-regime (all *p* < 0.0001) combinatorial treatments significantly improved survival compared to mock, no significant difference among cisplatin plus Δ*M062R* (median survival 96 days), cisplatin plus DC (median survival 113 days), and cisplatin plus Δ*M062R* and DC vaccine (median survival 115 days) was observed, suggesting the importance of early cisplatin treatment in survival. Adding Δ*M062R* (*p* = 0.0002) or DC vaccine (*p* < 0.0001) to cisplatin significantly improved treatment outcome compared to cisplatin alone. Clustered Δ*M062R* treatment (four consecutive doses every two days) at an early time (7 days post-tumor establishment) followed by clustered DC vaccine treatment (four consecutive doses every week) showed no difference in survival compared to DC vaccine alone starting at 7 days. This prompted us to investigate utilizing individual Δ*M062R* and DC vaccines sequentially in a combination treatment to exploit the immunostimulatory properties of Δ*M062R* in facilitating the effects of the DC vaccine, which is shown in the next section.

### 3.5. Scheduling of ΔM062R MYXV Treatment Is Critical to Achieve the Optimal Immunotherapeutic Outcome

**Figure 5:**
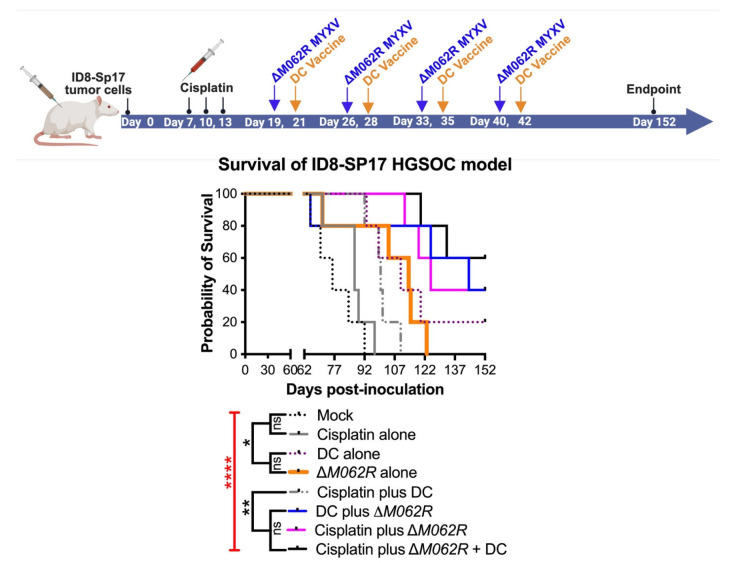
**Treatment with Δ*M062R* provides a survival advantage in the HGSOC model with antigen-targeted immunotherapy.** (**A**). Treatment design. C57/BL6 mice were injected i.p. with 1 × 10^6^ ID8-Sp17 cells at day 0. Animals were randomly assigned to each treatment group with 4 mice per group. Cisplatin, in single or combinatorial treatment, was injected i.p. at 1 mg/Kg starting at 7 days post-tumor establishment for a total of 3 treatments every 3 days. In the cisplatin-ΔM062R-DC or ΔM062R-DC combination treatments, ΔM062R-DC was administered 2 days before each DC vaccine. The ΔM062R-DC treatment block is repeated 4 times every 7 days. In treatments where Δ*M062R* or DC is not coincident, the Δ*M062R* or DC treatment was given on the set days shown in the diagram. (**B**). Kaplan–Meier analysis of outcomes showed the therapeutic benefit of ΔM062R. The log-rank (Mantel–Cox) test was conducted with statistical significance defined as the following, * *p* < 0.05, ** *p* < 0.001, **** *p* < 0.0001, and ns: not significant.

To further investigate the immunotherapeutic potential of Δ*M062R*, we revised our experimental system to utilize OC ID8 cells engineered to overexpress Sp17 tumor antigen [26], designated ID8-Sp17. In this model system, we specifically investigate how Δ*M062R* impacts Sp17-targeted DC vaccine when Sp17 expression is elevated in tumor cells. We also reduced the dose of cisplatin from 3 mg/Kg to 1 mg/Kg, as it was shown that low dose cisplatin possessed immunotherapeutic benefit while maintaining its chemotherapeutic effect [47]. We tested if Δ*M062R* presents an immunostimulatory effect directly to the sequential DC vaccine as one integrated treatment course. As shown in Figure 5 top diagram, the three-regime combinatorial treatment, 6 days after the completion of cisplatin, Δ*M062R* virotherapy is followed by DC vaccine 2 days later, and this Δ*M062R*-DC vaccine sub-regime is repeated weekly four times. Besides the mock treatment group, single-agent or dual-agent regimens are scheduled in a fixed time frame and frequency as shown in Figure 5 top diagram for the corresponding agent, such as the cisplatin-Δ*M062R*-DC vaccine regimen. We found that significant survival differences were observed (Kaplan–Meier survival and Long-rank Mandel–Cox test *p* < 0.0001) (Figure 5 bottom, survival curve). Treatment with DC alone (median survival 110 days, *p* = 0.0018) and Δ*M062R* alone (median survival 114 days, *p* = 0.0197) significantly improved survival compared to mock treatment (median survival 76 days) and cisplatin-only treatment (median survival 87 days) with *p* = 0.0066 and *p* = 0.0277 for DC alone and Δ*M062R* alone, respectively. Interestingly, we did not see significant differences in survival among Δ*M062R* plus DC (median survival 144 days), cisplatin plus Δ*M062R* (median survival 125 days), and the triple combinatorial regime (median survival undefined). However, these 3 treatments performed significantly better than cisplatin plus DC (median survival 100 days, Δ*M062R* plus DC *p* = 0.0342, cisplatin plus Δ*M062R p* = 0.0018, and triple-regime *p* = 0.0018). Because of the relatively small sample size (*n* = 4 per group), we did not see a significant difference between DC alone and cisplatin plus DC or Δ*M062R* plus DC, but the longer median survival time by treatment of Δ*M062R* plus DC reflected a trend of better survival than that by DC alone or by cisplatin plus DC. We thus concluded that to achieve a superior immunotherapeutic response, Δ*M062R* virotherapy should be placed prior to DC vaccine as a sub-regime. If low dose cisplatin is chosen for therapy, providing either Δ*M062R* or DC vaccine will lead to significantly improved survival, with Δ*M062R* (median survival 144 days) a better choice than DC vaccine (median survival 100 days). Overall, Δ*M062R* with DC drastically improved low-dose cisplatin to achieve relatively long-term survival (median survival undefined).

### 3.6. M062R-Null MYXV Infection of Ovarian Cancer Patient Ascites CD14^+^ Cells Improved CD4^+^ T Cell Anti-Tumor Response in a Primary Cell Co-Culture System

**Figure 6:**
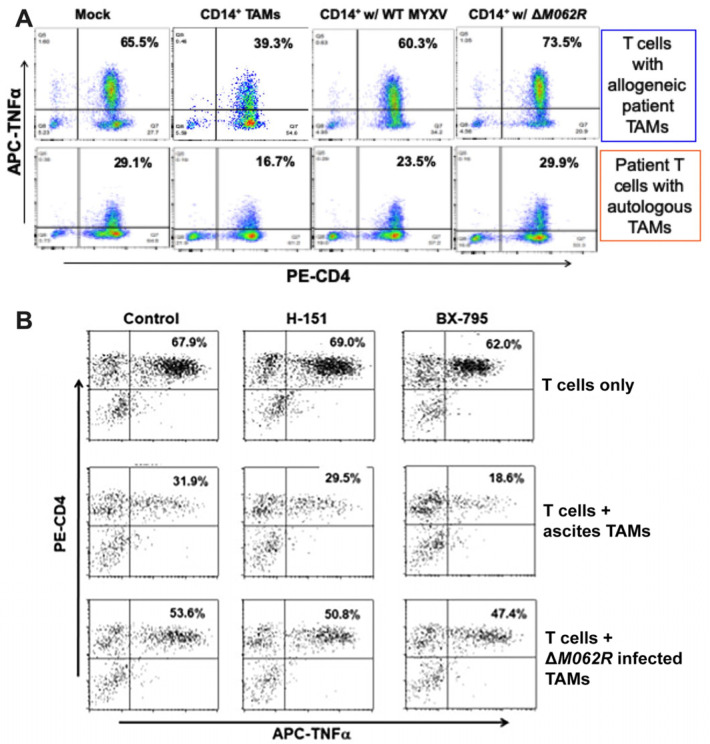
**Myxoma virus treatments to OC CD14^+^ TAMs promote CD4^+^ T cell activation against tumor antigen.** A co-culture system with TADG14v peptide tumor antigen-specific CD4^+^ T cells and OC ascites CD14^+^ TAMs was used for this study. (**A**). MYXV treatments of CD14^+^ TAMs reversed the immunosuppressive effect by TAMs. After incubating without any TAMs (mock), with untreated TAMs, with WT MYXV treated TAMs, or with Δ*M062R* treated TAMs, TNFa expression by allogeneic (top row) or autologous (bottom row) CD4^+^ T cells was determined following overnight stimulation with tumor peptide antigen-loaded autologous lymphoblastoid cells. A representative result from 3 individual patient TAM samples is shown. (**B**). Reversal of TAM immunosuppression by Δ*M062R* is independent of the cGAS/STING/TBK1/IRF3 signaling axis. CD4^+^ T cell functionality was measured as described above in (**A**). A STING antagonist, H-151 (1 μM), and a TBK1 kinase inhibitor, BX-795 (2 μM), were used to inhibit different steps of the DNA sensing pathway in cultures of T cells alone (first row), T cells plus CD14^+^ TAMs (second row), or T cells plus Δ*M062R* MYXV-treated TAMs (third row).

It is known that MYXV favors infecting monocytes/macrophages [48], providing a mechanistic rationale of targeting myeloid cells for immunotherapy with these viral agents. Based on our prior finding on the potent immunostimulatory effect of Δ*M062R* in monocytes and macrophages [18,43], we further examined whether the immunotherapeutic benefit of Δ*M062R* is due to its effects on the immunosuppressive tumor-associated monocyte/macrophage population in the tumor environment, designated immunosuppressive CD14^+^ tumor-associated macrophages (TAMs). We thus utilized a highly innovative co-culture system to test how OC patient CD4^+^ T cell functions are regulated in OC tumor environment. It is known that in OC CD14^+^ TAMs suppress the anti-tumor function of CD4^+^ T cells [34]. The key question is whether MYXV influences or even subverts immunosuppression by CD14^+^ TAMs to enable a more effective anti-tumor CD4^+^ T cell response. In this co-culture system, patient CD4^+^ T cells were stimulated with tumor antigen (TADG14v) [34] pulsed DC in the presence of TAMs with or without prior treatments. We then examined the status of CD4^+^ T cell activation reflected by TNF production via flow cytometry [34]. As shown in Figure 6A, in the absence of CD14^+^ TAMs, human CD4^+^ T cells responded to tumor antigen with TNF production (Figure 6A, first column). In the presence of TAMs, however, upon the same stimulation of OC tumor antigen, CD4^+^ T cells presented a significant reduction in TNF production (Figure 6A, second column). Both WT and Δ*M062R* treatments of TAMs reversed the immunosuppressive effect by TAMs, but with Δ*M062R* treatment leading to promotion of T cell activation. Shown in Figure 6A (top panel) is a representative result from an allogeneic co-culture system with CD4^+^ T cells and CD14^+^ TAMs from different donors. We also performed similar tests using autologous specimens for the co-culture system and achieved similar results (shown in Figure 6A, bottom panel).

Our previous study showed that Δ*M062R* activated CD14^+^ monocytes/macrophages to promote proinflammatory responses regulated by both DNA-sensing via the cGAS/STING/TBK1/IRF3 axis and the SAMD9 pathway [18]. Using a STING antagonist, H151, and a TBK1 inhibitor, BX-795, we found that reversal of TAM immunosuppression by Δ*M062R* was not affected (Figure 6B). We thus conclude that the therapeutic effect of Δ*M062R* against immunosuppressive TAMs is independent from the cGAS/STING/TBK1/IRF3 axis and is likely determined by the SAMD9 pathway. This is consistent with our observation that Δ*M062R* treatment induced a unique transcriptional landscape and cellular translation profile independent from the DNA-sensing pathway [43].

## 4. Discussion

MYXV was first formulated as an oncolytic agent almost 2 decades ago [49] and has been examined for its therapeutic potential against multiple cancer types [50,51,52,53]. The WT MYXV and replication competent MYXVs armed with therapeutic molecules have been developed to purge metastatic [16,51] and hematological malignant cells [54,55,56] as well as being loaded on vehicle cells for a trojan-horse like delivery [19,57]. Furthermore, MYXV can be used in coordination with chemotherapeutic agents to achieve improved treatment outcomes in preclinical models [20,21,58,59]. Our previous study found that replication-competent WT MYXV provided better therapeutic benefit in an OC carcinomatosis model when it was used before cisplatin treatment [20]. Interestingly, a replication-defective Δ*M062R* MYXV achieved superior therapeutic benefit as a monotherapy and as an adjuvant with cisplatin in combatting OC carcinomatosis [20]. In contrast with WT MYXV, the optimal schedule for Δ*M062R* MYXV is to be used after cisplatin treatment [20]. Infection by Δ*M062R* does not lead to progeny viral production [27,60]; thus, treatment with Δ*M062R* does not cause amplifying oncolysis seen with WT MYXV. Our recent work demonstrated that Δ*M062R* infection triggered proinflammatory cytokine/chemokine production in monocytes/macrophages [18]. Such a proinflammatory response is triggered in part through the DNA-sensing pathway leading to IFN-I production [18]. However, upon transcriptomic analyses of the effect of Δ*M062R* on monocytes/macrophages, a distinct feature from that of the cGAS/STING/TBK1/IRF3 axis of the DNA sensing pathway was observed in monocytes/macrophages by Δ*M062R* [43]. This unique response is likely due to the activation of another host immunoregulatory factor, SAMD9, that is the host target of the viral M062 protein [27,61]. Activating the SAMD9 pathway by Δ*M062R* is associated with the immunotherapeutic potential of this replication-defective viral agent. Although the concept of the immunotherapeutic potential of oncolytic viruses has been recognized [62], the therapeutic promise of a replication defective viral vector is not yet broadly recognized. In this study, we presented strong evidence that such a replication-defective Δ*M062R* MYXV can enhance immunotherapy for OC and alleviate ovarian TAM immunosuppression of DC-stimulated tumor antigen-specific human CD4^+^ T cell responses. The MYXV Δ*M062R* platform is an excellent option for immunotherapy in OC and can be utilized as an adjuvant to existing chemotherapy and DC vaccine.

The utility of Δ*M062R* in immunotherapy is due to its immunostimulatory property by activating DNA-sensing-associated IFN-I induction and the SAMD9 pathway [18,43]. However, the timing of Δ*M062R* treatment is important to achieve therapeutic benefit. We found that early treatment with Δ*M062R* (1 week after tumor cell inoculation) did not render significant benefit, nor did it enhance the benefit of the DC vaccine (Figure 4B,E). On the other hand, when Δ*M062R* monotherapy was provided much later (a month after tumor cell inoculation), it outperformed cisplatin monotherapy that started 2 weeks before Δ*M062R* treatment (Figure 3B). Moreover, Δ*M062R* treatment following chemotherapy in a combination regimen preceding DC vaccine (Figure 3B and Figure 4E) or Δ*M062R* given alone preceding DC vaccine (Figure 5) provided superior survival benefit over chemotherapy alone, suggesting that Δ*M062R* may bring added benefit to DC vaccination in the clinical setting for OC patients.

Among human leukocytes, MYXV presents preferred binding and entry in myeloid cells such as monocytes and macrophages [48], which establishes the rationale of using this viral vector against TAMs. To investigate possible mechanisms of Δ*M062R*-DC vaccine combinatorial treatment, we examined how Δ*M062R* influenced monocytes/macrophages to activate innate immune signaling events [18,43]. In OC TAMs, Δ*M062R* likely silences the expression of immunosuppressive cytokines/chemokines [20] or even stimulates production of proinflammatory molecules. Alleviation of immunosuppression by CD14^+^ TAMs allows stronger CD4^+^ T cell responses to tumor antigen stimulation (Figure 6A). Besides being an informative predictor for prognosis [63], the immunosuppressive properties of the OC tumor environment also affect the efficacy of immunotherapy agents, including DC vaccine [64] and CAR-T therapy [65,66]. The immunotherapeutic benefit of Δ*M062R* may not be restricted to monocytes/macrophages, as enhancement of T cell responses by MYXV has also been reported [67]. MYXV hijacks cells with short-circuited IFN-I responses, often malignant cells or cells cultured in the tumor environment [54], but does not affect the functionality of normal cells such as hematopoietic stem cells [54,68], progenitor cells [54,68], and normal T cells [67,69], suggesting a favorable safety profile of Δ*M062R* for clinical applications in the future.

## 5. Patents

A US patent resulting from the work reported in this manuscript, US 12,128,099 B2, was awarded to J.L. and M.J.C.

## Figures and Tables

**Table 1:** RT-PCR primers.

Target Gene	Primer Sequences
Human IFNβ	Fwd 5′-GCC ATC AGT CAC TTA AAC AGC-3′
	Rev 5′-GAA ACT GAA GAT CTC CTA GCC T-3′
Human IL-12b	Fwd 5′-CAAAGGAGGCGAGGTTCTAA-3′
	Rev 5′-GCAGGTGAAACGTCCAGAATA-3′
Human Sp17	Fwd 5′-GGTTCCATAGGCAGTTCTTAC-3′
	Rev 5′-GGAAGGCAGCTTGGATTT-3′
Human RSAD2	Fwd 5′-AGT GCA ACT ACA AAT GCG GC-3′
	Rev 5′-CTT GCC CAG GTA TTC TCC CC-3′
Human CXCL-10	Fwd 5′-CTG TAC CTG CAT CAG CAT TAG TA-3′
	Rev 5′-GAC ATC TCT TCT CAC CCT TCT TT-3′
Human IL15	Fwd 5′-AGCCAACTGGGTGAATGTAATA-3′
	Rev 5′-CATCTCCGGACTCAAGTGAAATA-3′
Human ISG54	Fwd 5′-AGCGAAGGTGTGCTTTGAGA-3′
	Rev 5′-GAGGGTCAATGGCGTTCTGA-3′
Human NFκB1A	Fwd 5′-CCCTACACCTTGCCTGTGAG-3′
	Rev 5′-TGACATCAGCACCCAAGGAC-3′
Human CCL3	Fwd 5′-CTCTCTGCAACCAGTTCTC-3′
	Rev 5′-CTGCTCGTCTCAAAGTAGTC-3′
Murine Sp17	Fwd 5′-CTTTCTCCAACACCCACTAC-3′
	Rev 5′-CTTCATCTTCTTTACCTCTTCTCT-3′
Murine IL-10	Fwd 5′-AGGCGCTGTCATCGATTTCT-3′
	Rev 5′-ATGGCCTTGTAGACACCTTGG-3′
Murine CD40	Fwd 5′-GTAGGTCACCCCTGAGAACC-3′
	Rev 5′-ACAACCCGAACCATACACACAA-3′
Murine CX3CL1 [29]	Fwd 5′-GCTCCTAGCCCTGACCCATC-3′
	Rev 5′-AGCTGATAGCGGATGAGCAA-3′
Murine GM-CSF	Fwd 5′-CTGGCCCCATGTATAGCTGA-3′
	Rev 5′-ACAGTCCGTTTCCGGAGTTG-3′
Murine IL-6	Fwd 5′-TCAATATTAGAGTCTCAACCCCCA-3′
	Rev 5′-GAAGGCGCTTGTGGAGAAGG-3′
Murine iNOS [30]	Fwd 5′-ATCGACCCGTCCACAGTATG-3′
	Rev 5′-GATGGACCCCAAGCAAGACT-3′
Murine TNFα	Fwd 5′-CCCTCACACTCACAAACCAC-3′
	Rev 5′-ACAAGGTACAACCCATCGGC-3′
Murine IFNβ	Fwd 5′-AGATCTCTGCTCGGACCACC-3′
	Rev 5′-CGTGGGAGATGTCCTCAACT-3′
Murine CXCL10	Fwd 5′-ATGACGGGCCAGTGAGAATG-3′
	Rev 5′-TCGTGGCAATGATCTCAACAC-3′
Murine IRF3.2	Fwd 5′-CACTCCCCACGCTACACTC-3′
	Rev 5′-TCCCATCCCCAGTAGCATGAG-3′
Murine IRF7.2 [31]	Fwd 5′-TGCTGTTTGGAGACTGGCTAT-3′
	Rev 5′-TCCAAGCTCCCGGCTAAGT-3′
Murine IFNγ	Fwd 5′-CGGCACAGTCATTGAAAGCC-3′
	Rev 5′-TGTCACCATCCTTTTGCCAGT-3′
Murine CXCL1 [29]	Fwd 5′-GCTGGGATTCACCTCAAGAA-3′
	Rev 5′-TCTCCGTTACTTGGGGACAC-3′
Murine CXCL3 [32]	Fwd 5′-CCACTCTCAAGGATGGTCAA-3′
	Rev 5′-GGATGGATCGCTTTTCTCTG-3′
Murine MCP-1 [30]	Fwd 5′-AGGTCCCTGTCATGCTTCTG-3′
	Rev 5′-GGATCATCTTGCTGGTGAAT-3′
Murine EGR1	Fwd 5′-CACCTGACCGCAGAGTCTTTT-3′
	Rev 5′-GCGGCCAGTATAGGTGATGG-3′

## Data Availability

No additional data besides what are shown is included in the study.

## Abbreviations

The following abbreviations are used in this manuscript:

MYXV	myxoma virus
OC	ovarian cancer
DC	dendritic cell
TAM	tumor-associated macrophage
HGSOC	high-grade serous ovarian cancer
